# Mapping the research hotspots and trends in midwifery education: A bibliometric analysis

**DOI:** 10.1097/MD.0000000000049776

**Published:** 2026-07-17

**Authors:** Nuray Kurt, Osman Tayyar Çelik, Esra Sabanci Baransel, Tuba Uçar

**Affiliations:** a Department of Midwifery, Faculty of Health Sciences, Firat University, Elaziğ, Türkiye; b Department of Child Development, Faculty of Health Sciences, İnönü University, Malatya, Türkiye; c Department of Midwifery, Faculty of Health Sciences, İnönü University, Malatya, Türkiye.

**Keywords:** bibliometrics, education, mapping, midwifery

## Abstract

**Introduction:**

Midwifery, which used to be practiced with knowledge passed down from generation to generation, has now gained a professional identity through education. This study examines global trends in midwifery education research using bibliometric methods.

**Methods:**

In the study, we analyzed 1029 studies published in the Web of Science database on midwifery education between 1993–2023. Bibliometric and visualization analyses revealed the general structure of the literature by determining important trends, thematic research areas, the most influential publications, journals, and countries in midwifery education.

**Results:**

According to the results of the analysis, studies on midwifery education have increased significantly in recent years. The increase in publications appears to coincide with the growing prominence of topics such as digitalization, simulation-based learning, and distance education in the literature. Australia stands out as one of the most influential and productive countries in the field of midwifery education. In addition, the continuing care model and simulation-based learning in midwifery were among the most frequently studied topics.

**Discussion:**

The study identifies research trends, priority research areas, and areas for development in midwifery education. The findings provide important insights for the development of midwifery education programs, the support of policymakers’ decision-making processes, and the identification of future research priorities.

## 1. Introduction

Midwifery, which used to be practiced with knowledge passed down from generation to generation, has now gained a professional identity through education.^[1]^ Competent midwives make a difference in the lives of women and newborns.^[2]^ Midwives who receive high-quality standard training have been identified as having a key role in reducing maternal and neonatal deaths and stillbirths, saving 4.3 million lives annually by 2035.^[3]^ Midwifery, which includes proven interventions for maternal and neonatal health as well as family planning, “can prevent more than 80% of all maternal deaths, stillbirths and neonatal deaths.”^[4]^ Therefore, high-quality midwifery education programs are vital to training competent midwives who can provide high standards of safe, evidence-based care to women and newborns.^[3]^

Midwifery education is the basis for equipping midwives with appropriate competencies to provide safe, evidence-based care to women at a high standard.^[2]^ Undergraduate education is of great importance in terms of midwives being able to respond to the health needs of women, babies, families, and society, and strengthening their professional identity.^[1]^ When midwives are trained, licensed, regulated according to international midwifery standards, and fully integrated into a well-functioning health system and an interprofessional team with referral services when necessary for emergencies, they can provide the full range of interventions needed. Strengthening midwifery education according to international standards is an important step for improving the quality of care and reducing maternal and neonatal deaths and morbidities.^[4]^ To increase the professional strength of the profession, there is a need for the determination of “National and International” ethical standards, as well as educational standards that provide specialized professional knowledge and skills. Qualified education and educational elements play an important role in meeting these needs.^[1]^

In recent years, digitalization, distance learning, simulation-based education, and AI-supported applications have led to significant transformations in the education of health professions. These developments have also impacted midwifery education, contributing to the emergence of new research areas related to teaching methods, learning environments, and the assessment of student competencies. This rapid transformation necessitates a more comprehensive examination of research trends and the knowledge structure within the field of midwifery education.^[5,6]^ However, this process of transformation and knowledge production does not unfold in the same way across all countries. In particular, limitations regarding educational infrastructure, academic resources, and research capacity in low- and middle-income countries can lead to significant disparities in knowledge production and scientific visibility. This situation necessitates a more holistic assessment of research priorities in midwifery education and the global knowledge production structure.^[3,4]^

As in other fields, there are systematic reviews and meta-analysis studies based on the reexamination of previous research in the field of midwifery education.^[5–7]^ However, little is known about the hot spots of research in the field of midwifery education and the future directions of this field. Especially in recent years, the increase in the number of scientific publications has made it difficult to examine the literature with traditional methods. As another method, there has been a rapid increase in the number of bibliometric studies aimed at revealing the general appearance, structure, and trends of scientific disciplines and studies on specific topics in recent years.^[8]^ Bibliometric methodologies are also quite suitable for determining journal performance, co-authorships, co-citation trends, and also classical research streams of specific fields.^[8,9]^ Bibliometric-based studies also offer many added values to the literature in terms of being able to make a retrospective analysis of a subject, to determine in which direction the field is expanding, to understand the general structure, and to provide a comprehensive photograph from different perspectives.^[10]^ While traditional reviews make significant contributions to synthesizing evidence related to specific research questions, they may be limited in their ability to reveal the intellectual structure, research networks, thematic development, and patterns of knowledge production within a broad and rapidly expanding body of literature. Therefore, bibliometric analyses enable a more comprehensive assessment of a research field’s developmental trajectory, knowledge structure, and research gaps.^[8–12]^

Since it has been the subject of scientific studies for many years, the literature on midwifery education has developed considerably. However, there are no studies that present a comprehensive picture of the international literature on midwifery education. In this context, the research aims to review the basic and conceptual structure of the current research field on midwifery education from a macro perspective using bibliometric methods. Furthermore, the following questions were addressed by focusing on a wide set of documents covering the international literature:

1. What is the distribution of articles in the field of midwifery education by year?
2. Which are the most influential publications, authors, journals, institutions, and countries in the midwifery education literature?
3. What are the trending study topics in the field of midwifery education?
4. How is the co-authorship cooperation of researchers?
5. What are the themes that emerge in the field of midwifery education according to content analysis?

## 2. Methods

### 2.1. Study design

In the study, the bibliometric method was used to examine the publication outputs related to midwifery education and the basic structure of the field. The bibliometric method is quantitative and is a method that evaluates the performance of the literature in a certain field based on the number of publications and citations.^[11]^ This method provides a comprehensive understanding of the whole of the studies from a macroscopic perspective in literature reviews. Thus, it is possible to obtain information about the whole of the studies thanks to a macro view in literature reviews.^[12]^

### 2.2. Data collection andt search strategy

**Figure 1.**
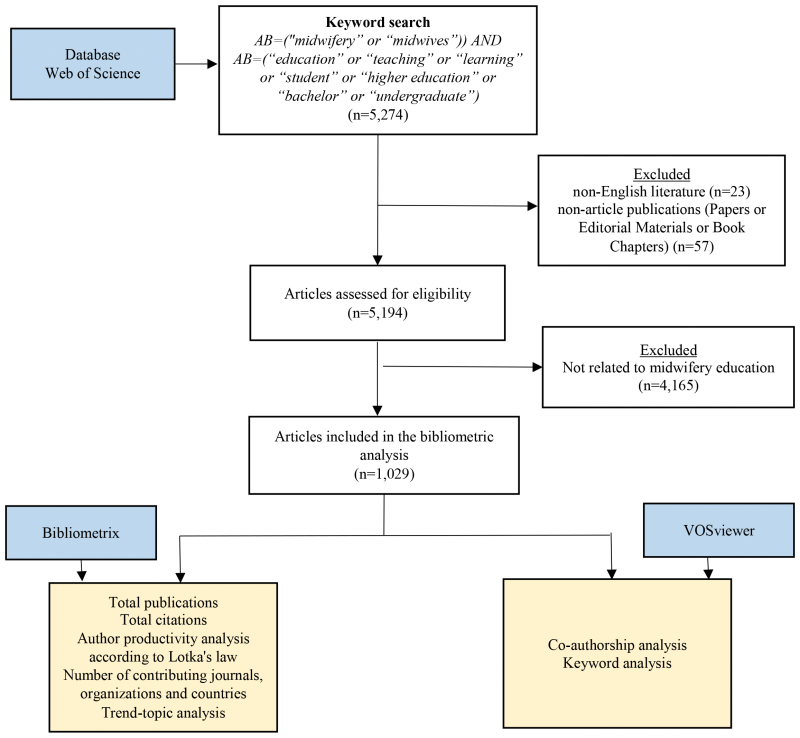
Research flow diagram.

The research flow diagram showing the data collection and scanning strategies is given in Figure 1. In the research, the Web of Science (WoS) database was used as a data source. With over 171 million records, WoS is one of the most widely used citation databases, providing comprehensive bibliographic and citation data on scientific publications. These features enable the detailed analysis and classification of scientific publications.^[13]^

In this study, the WoS database was selected because it provides high-quality, standardized bibliographic records; is widely used in bibliometric analyses; and offers reliable data for citation analyses, co-authorship networks, and keyword co-occurrence maps. Furthermore, using a single database enhances the consistency of analyses by reducing duplicate records and metadata inconsistencies that may arise when multiple databases are used together.^[13]^

During the development of the search strategy, previous studies and relevant reviews in the field of midwifery education were reviewed. Keywords were identified through discussion by the research team and refined to include publications related to midwifery education. Before the final search strategy was implemented, the research team tested various keyword combinations and evaluated whether the resulting records were relevant to the study topic. Based on these evaluations, the search terms were refined, and the final search strategy was developed (Fig. 1).

The publications were scanned using the advanced search strategy in the WoS database with these keywords. As a result of the scan performed without any restrictions on January 17, 2024, 5274 studies were reached. Then, filtering was applied, and the document type was the article, the index was SSCI, SCI-E, ESCI, AHCI, and the publication language was English. As a result of this filtering, 5194 studies remained. The titles and abstracts screening was conducted independently by 2 researchers (ESB and NK). The researchers evaluated all records individually according to predefined inclusion and exclusion criteria. Disagreements that arose during the evaluation process were resolved through consensus following discussions among the researchers. As a result of this process, 4165 studies unrelated to the scope of the research were excluded, and 1029 studies were identified for inclusion in the analyses.

The inclusion criteria were: studies that included only midwifery students in the sample, and studies that included other health professionals/students in addition to midwifery students in the sample. The exclusion criteria were: studies that included only nursing or other health sciences students in the sample, studies that evaluated in-service training given to midwives, studies that included only midwife educators in the sample, and studies that included pregnancy education (such as antenatal education, childbirth education). The screening resulted in 1029 studies covering the years 1993–2023 (Fig. 1). Since this study is based solely on the analysis of published bibliographic records in the WoS database, it does not require approval from an ethics committee or informed consent.

### 2.3. Data analysis

The metadata of 1029 articles accessed from the WoS database were downloaded in plain text format and loaded into bibliometric analysis programs. Bibliometrix (version 4.1.2; https://www.bibliometrix.org) and VOSviewer (version 1.6.9; www.vosviewer.com) were used for bibliometric analysis. Bibliometrix was written in the R programming language. After the library (“bibliometrix”) code is loaded via RStudio, the Bibliometrix web interface is opened with the biblioshiny() command. The Bibliometrix package facilitates both performance analysis and scientific mapping.^[14]^ VOSviewer is a software that creates and visualizes maps based on network data.^[15]^

In bibliometric analyses, the number of publications and the number of citations are among the key performance indicators. In this context, the distribution of publications by year, journal, institution, and country was examined using frequency analysis. In the citation analyses conducted to identify the most influential publications and journals in the field, the number of local citations was taken into account. The number of local citations refers to citations between the studies included in the analysis. This metric allows for a clearer understanding of the knowledge structure within the field and the foundational studies that shape it.^[14]^ To identify the countries producing the most publications, the country of the corresponding author was used.

Lotka Law was used to assess authors’ productivity levels. This analysis examined the publication productivity of authors who have contributed to the literature on midwifery education.

Keyword analyses were conducted to identify the current research topics in the field and the conceptual framework. In this context, author keywords were used in the analyses instead of Keywords Plus. Since author keywords are directly determined by the researchers, they more accurately reflect the core content and conceptual focus of the studies. In contrast, because Keywords Plus is generated algorithmically, it may include concepts that do not directly represent the study’s central theme.

Prior to the keyword analysis, a synonym file in txt format was created for synonymous terms. As part of keyword normalization, different terms expressing the same concept were consolidated under a single concept. For example, the phrases “midwifery student,” “midwifery students,” and “student midwives” were evaluated under the same concept. This process contributed to reducing conceptual redundancy and more accurately revealing the thematic structure of the field.^[16]^

Subsequently, a co-occurrence analysis was conducted, and the resulting clusters were thematically classified by the researchers based on the common conceptual focus of the dominant keywords they contained. Conceptually related keywords were grouped under broader themes, and the resulting themes were finalized following discussions among the researchers.

Finally, a co-authorship analysis was conducted to examine scientific collaboration between countries. Co-authorship analysis is a widely used method for assessing the structure of academic interaction and collaboration among researchers.^[17]^

## 3. Results

**Figure 2.**
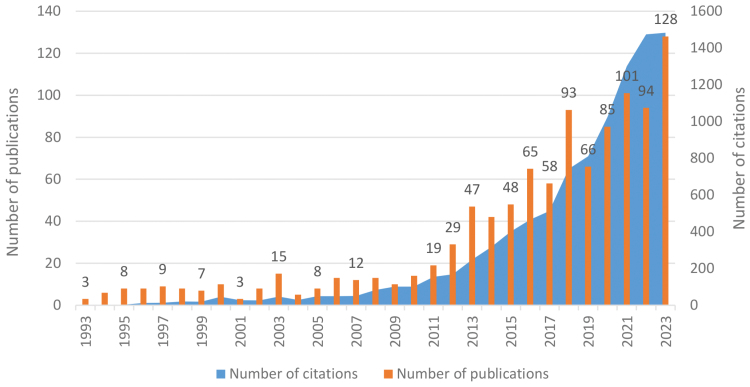
Annual distribution of publications and citations in the midwifery education literature.

Based on the search strategy, a total of 1029 articles were obtained and included in the study until the end of 2023 in midwifery education. Figure 2 presents the production dynamics and citations of the literature on midwifery education. Among them, there were 925 research articles (89.9%) and 104 reviews (10.1%). The first publication was published in 1993. It is seen that there was a rapid increase in publications after 2011. In this context, the years between 1993 and 2011 can be characterized as the formation phase of the midwifery education research base. An accelerated increase is observed between 2011 and 2023. Therefore, these periods can be evaluated as the development phase of the research field. It is possible to say the same about citations (Fig. 2).

**Figure 3.**
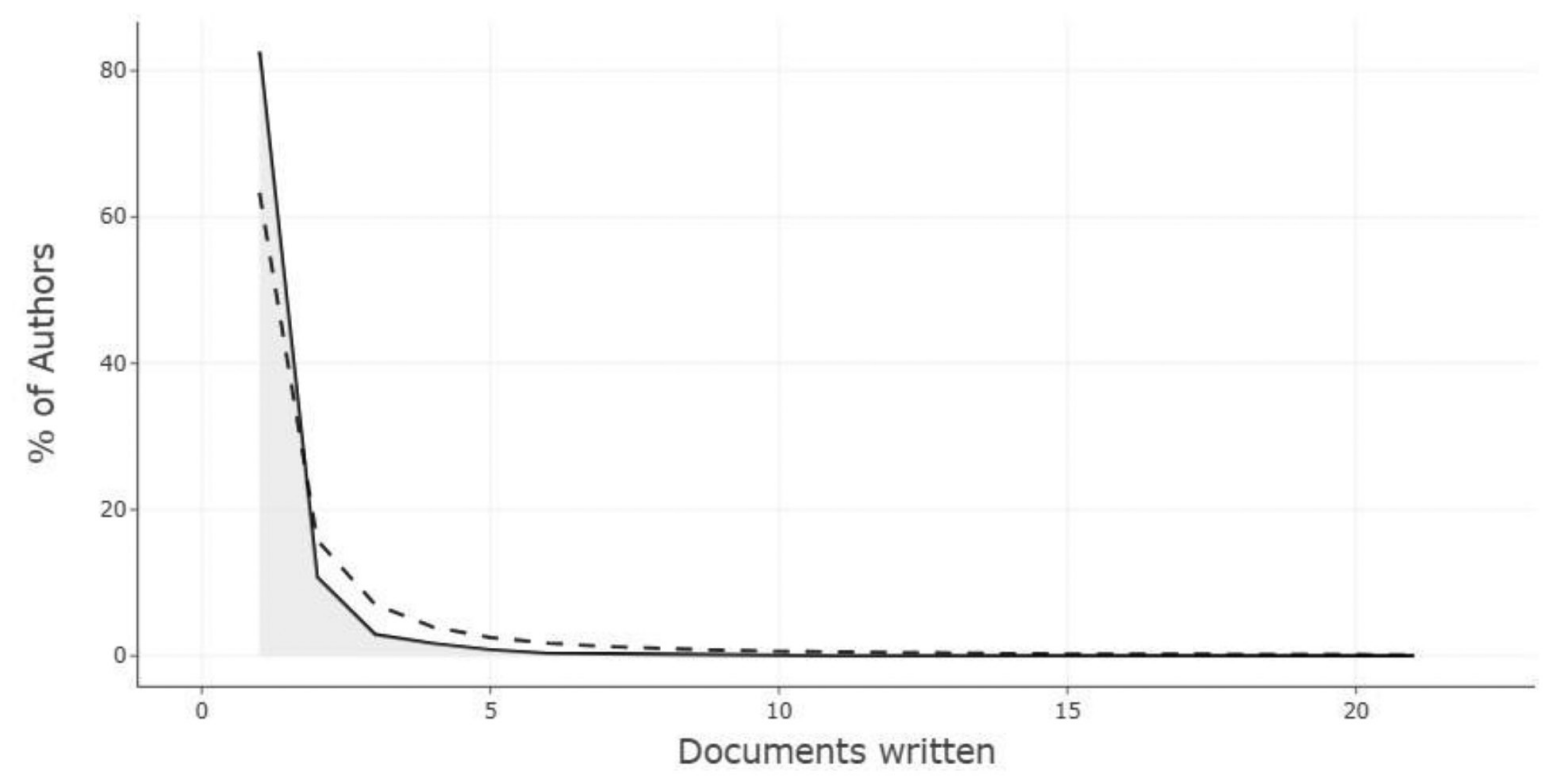
Distribution of authors publishing on midwifery education according to Lotka law.

The contribution of authors working in the field of midwifery education to the literature and their scientific productivity was examined according to Lotka’s Law. The distribution of authors publishing on midwifery education according to Lotka’s law is presented in Figure 3. When the analysis results were examined, it was determined that the rate of authors with 1 publication on midwifery education was 82% (n = 2585), authors with 2 publications was 11% (n = 337), authors with 3 publications was 3% (n = 91), authors with 4 publications was 2% (n = 53), and the rate of authors with 5 or more publications was 2% (n = 66).

Table 1 lists the 10 most frequently cited studies, comprising 4 qualitative studies, 3 research articles, 2 review articles, and 1 systematic review. Eight of these studies were published in Midwifery. Four of the most frequently cited articles were qualitative studies. This finding suggests that qualitative studies are valued by other researchers and shape discussions in the field. One of the most frequently cited publications was a systematic review of 24 articles examining the evidence for simulation-based learning in midwifery education. Four of the other most frequently cited articles were studies evaluating different aspects of continuing care in midwifery education programs in Australia. The other 3 most frequently cited articles were studies examining the effectiveness of competency-based education in midwifery education. The fact that the most frequently cited articles were in specific areas suggests that there is a strong interest in these areas and that research in these areas has a high impact on academic circles and practice.

**Table 1:** Top ten cited studies in midwifery education.

Rank	Authors	Title	Design	Local citations	Research domains
1	Cooper S, Cant R, Porter J, et al Women Birth. 2012;25 (2):64–78. doi:10.1016/j.wombi.2011.03.004	Simulation-based learning in midwifery education: a systematic review.	Systematic review	27	Simulation-based learning
2	McLachlan HL, Newton M, Nightingale H, Morrow J, Kruger G. Midwifery. 2013;29 (9):1064–1072. doi:10.1016/j.midw.2012.12.017	Exploring the “follow-through experience”: a statewide survey of midwifery students and academics conducted in Victoria, Australia.	Cross-sectional	27	Continuity of care
3	Licqurish S, Seibold C. Midwifery. 2008;24 (4):480–489. doi:10.1016/j.midw.2007.05.001	Bachelor of Midwifery students’ experiences of achieving competencies: the role of the midwife preceptor.	Qualitative	25	Role of the midwifery preceptor in learning and development of competency
4	Gray J, Leap N, Sheehy A, Homer CS. Midwifery. 2013;29 (4):400–406. doi:10.1016/j.midw.2012.07.015	Students’ perceptions of the follow-through experience in 3 yr bachelor of midwifery programmes in Australia.	Qualitative	23	Continuity of care
5	Carter AG, Wilkes E, Gamble J, Sidebotham M, Creedy DK. Midwifery. 2015;31 (8):765–771. doi:10.1016/j.midw.2015.04.006	Midwifery students’ experiences of an innovative clinical placement model embedded within midwifery continuity of care in Australia.	Descriptive cohort	22	Continuity of care
6	Rawnson S. Midwifery. 2011;27 (6):786–792. doi:10.1016/j.midw.2010.07.004	A qualitative study exploring student midwives’ experiences of carrying a caseload as part of their midwifery education in England.	Qualitative	20	Role of the caseloading in practice
7	Fullerton JT, Thompson JB, Johnson P. Midwifery. 2013;29 (10):1129–1136. doi:10.1016/j.midw.2013.07.006	Competency-based education: the essential basis of pre-service education for the professional midwifery workforce.	Review	20	Competency-based education
8	Ebert L, Tierney O, Jones D. Nurse Educ Pract. 2016;16 (1):294–297. doi:10.1016/j.nepr.2015.08.003	Learning to be a midwife in the clinical environment; tasks, clinical practicum hours or midwifery relationships.	Review	20	Continuity of care
9	Hughes AJ, Fraser DM. Midwifery. 2011;27 (4):477–483. doi:10.1016/j.midw.2010.03.006	“There are guiding hands and there are controlling hands”: student midwives experience of mentorship in the UK.	Qualitative longitudinal cohort	19	Role of the mentor in practice
10	Licqurish S, Seibold C. Midwifery. 2013;29 (6):661–667. doi:10.1016/j.midw.2012.06.006	“Chasing the numbers”: Australian Bachelor of Midwifery students’ experiences of achieving midwifery practice requirements for registration.	Qualitative	19	Competency-based education

Table 2 lists the most influential journals in midwifery education literature according to the number of publications and citations. Among these, Nurse Education Today, which published 141 studies and was cited 1702 times in total, Midwifery, which published 121 studies and was cited 1292 times in total, and Nurse Education in Practice, which published 107 studies and was cited 890 times in total, were in the first place. The number of publications and citations in these journals was parallel. In addition, these journals were the foundational journals of midwifery education literature and led the growth of the midwifery education research field.

**Table 2:** The most influential journals of midwifery education research.

Journal	Number of publications	Journal	Number of citations
Nurse Education Today	141	Nurse Education Today	1702
Midwifery	121	Midwifery	1292
Nurse Education in Practice	107	Nurse Education in Practice	890
Women and Birth	78	Journal of Advanced Nursing	738
Journal of Midwifery & Womens Health	64	Women and Birth	623
BMC Medical Education	32	Medical Education	394
Journal of Advanced Nursing	22	Journal of Nursing Education	376
Journal of Education and Health Promotion	17	Medical Teacher	360
Journal of Nurse-Midwifery	17	Journal of Midwifery & Womens Health	355
Journal of Clinical Nursing	13	Journal of Clinical Nursing	292

The 10 most productive institutions out of a total of 941 are presented in Table 3. It was seen that 7 universities in the top 10 institutions were in Australia, and Monash University, also in Australia, was the most productive institution with 88 publications.

**Table 3:** The ten most productive organizations.

Organizations	Country	Number of publications
Monash University	Australia	88
Griffith University	Australia	62
The University of Queensland	Australia	35
Isfahan University of Medical Sciences	Iran	34
University of Technology Sydney	Australia	32
Newcastle University	United Kingdom	30
Western Sydney University	Australia	29
University of Michigan	United States	28
Flinders University	Australia	27
La Trobe University	Australia	26

**Figure 4.**
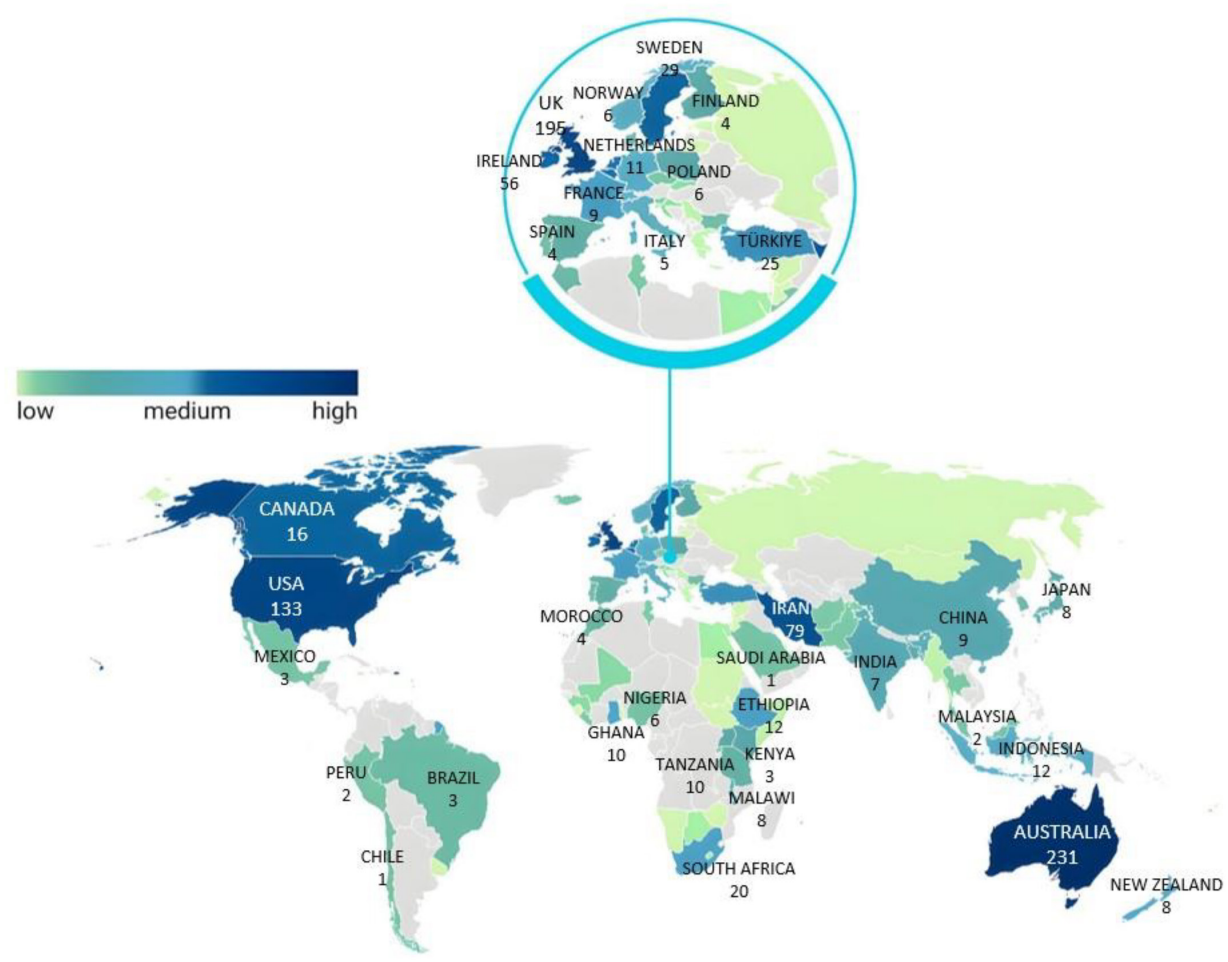
Scientific production by country based on corresponding author affiliation.

This distribution is based on referencing the country of the corresponding author in the articles. The corresponding authors produced 1029 publications from 66 countries. The most productive countries were Australia (n = 231, 22%) and the United Kingdom (n = 195, 19%). They were followed by the United States (n = 133, 12%), Iran (n = 79, 7%), and Ireland (n = 56, 5%) (Fig. 4).

**Figure 5.**
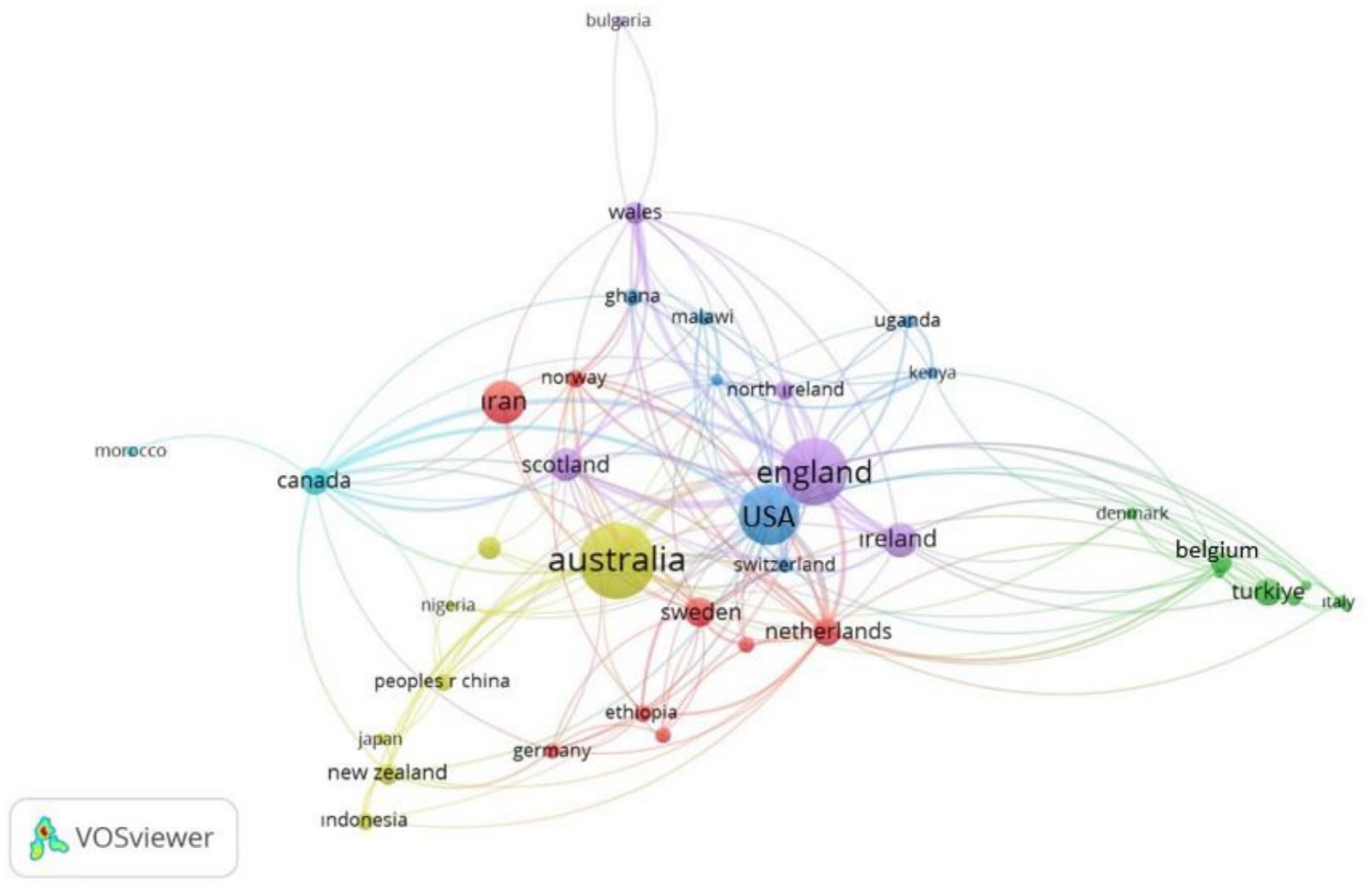
International collaboration network based on country co-authorship.

Cross-country collaboration based on co-authorship is shown in Figure 5. Each circle on the map represents a country, the size of the circle represents the number of published articles, and the lines between the circles represent the strength of collaboration among countries.^[18]^ The United Kingdom collaborated with 30 countries, the United States with 25 countries, and Australia with 22 countries. Except for Morocco, all other countries collaborated with 2 countries. The strongest collaboration occurred between Australia and the United Kingdom, which were also the 2 most productive countries.

**Figure 6.**
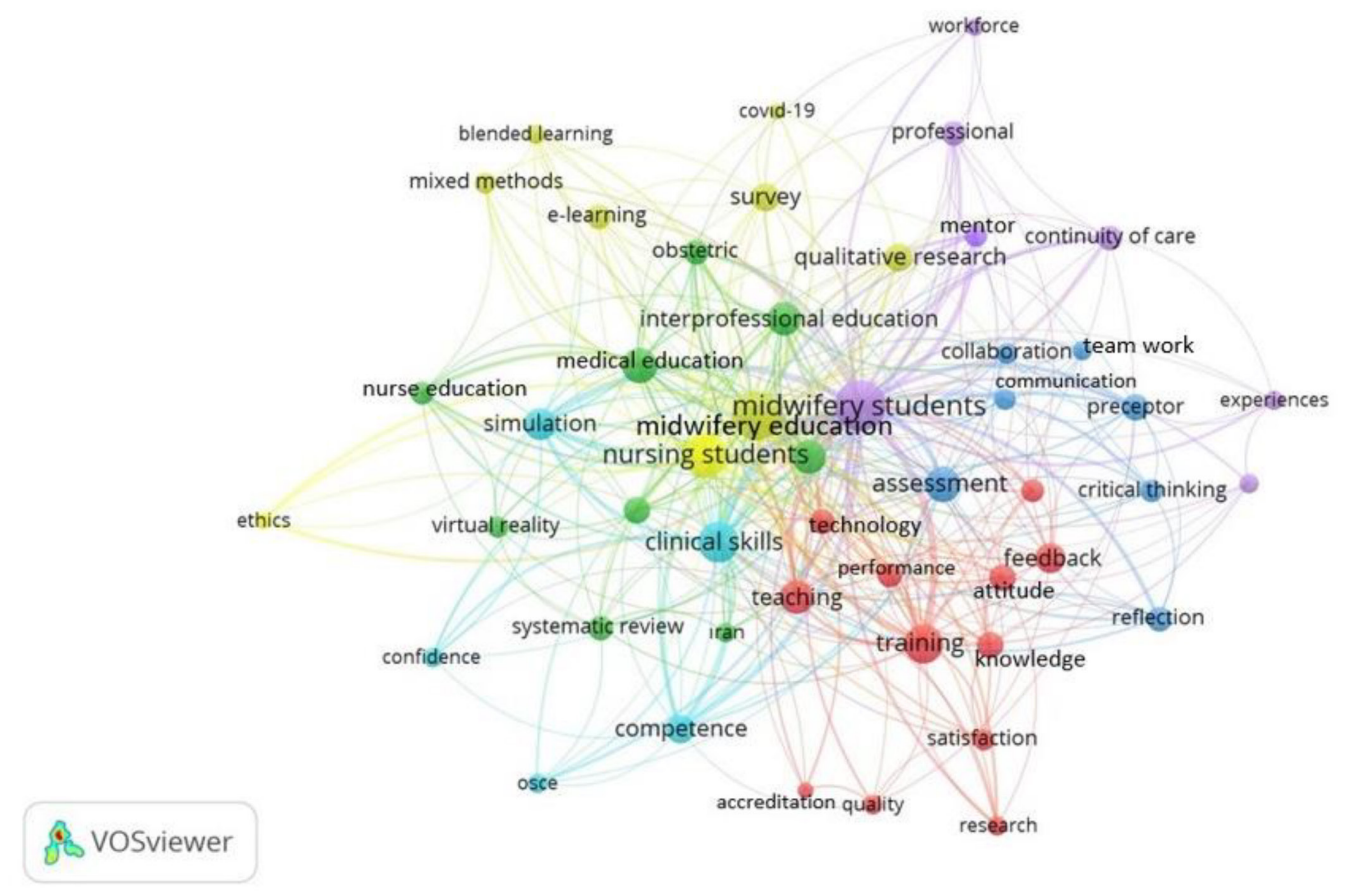
Keyword co-occurrence network based on author keywords.

A common keyword analysis was conducted using the author keywords in the studies included in the study (Fig. 6). Common keyword analysis visualizes the relationship between the variables examined together. Each node represents a keyword. Node size indicates the frequency of the keyword, colors represent thematic clusters, and lines indicate co-occurrence relationships between keywords.^[18]^ In the keyword co-occurrence analysis, the minimum occurrence threshold was set to 5 in order to identify the terms to be included in the network map. The aim was to focus on key concepts with sufficient visibility in the literature and to reduce the fragmented network structure that could result from low-frequency terms.

When the map was examined, it was seen that 49 keywords formed 6 different clusters. When the first 2 clusters with the highest number of keywords, the red cluster (cluster 1), are examined, it consists of 12 keywords; the most frequently used keywords are “training” (n = 53) and “teaching” (n = 25). The yellow cluster (cluster 2) consists of 9 keywords, and the most frequently used keywords are “midwifery education” (n = 316) and “nursing students” (n = 121). The green cluster (cluster 3) consists of 9 keywords, and the most frequently used keywords are “curriculum” (n = 33) and “interprofessional education” (n = 32). The blue cluster (cluster 4) consists of 7 keywords, and the most frequently used keywords are “assessment” (n = 33) and “preceptor” (n = 20). The purple cluster (cluster 5) consists of 7 keywords, and the most frequently used keywords are “midwifery students” (n = 466) and “mentor” (n = 30). The turquoise cluster (cluster 6) consists of 5 keywords, and the most frequently used keywords are “clinical skills” (n = 105) and “simulation” (n = 55) (Fig. 6).

The author keywords identified through cluster analysis were subjected to content analysis, and each cluster was thematically labeled based on the common conceptual focus of the dominant keywords it contained (Table 4). These themes are: quality of education and training, research in the field of midwifery education, education and training of health professionals, professional development and collaboration, professional development and support of midwifery students, development of clinical skills and simulation-based education.

**Table 4:** Research themes related to midwifery education.

Theme	Colour	The number of items	More frequent codes	Prevailing sub-categories
Quality of education and training	Red	12	Training (53), teaching (25), knowledge (11), technology (9), feedback (9), accreditation (8), research (8), satisfaction (8), attitude (7), breastfeeding (7), performance (7), quality (7)	Educational processes, information management, accreditation and standards, the role of technology in education
Research in the field of midwifery education	Yellow	9	Midwifery education (316), nursing students (121), qualitative research (24), e-learning (18), survey (13), ethics (11), covid-19 (9), blended learning (8), mixed methods (8)	Research methods and data collection in midwifery education, collaborations with nursing students, ethical issues and professional practices, pandemic and education, different learning techniques
Education and training of health professionals	Green	9	Curriculum (33), interprofessional education (32), nurse education (28), medical education (23), obstetric (21), nurse-midwives (20), iran (7), systematic review (7), virtual reality (7)	Curriculum development and innovative educational approaches, interdisciplinary education, systematic review, geographical focus (Iran)
Professional development and collaboration	Blue	7	Assessment (33), preceptor (20), reflection (17), critical thinking (9), teamwork (9), collaboration (8), communication (8)	Assessment and feedback, reflection and critical thinking, teamwork and collaboration, communication and effective communication skills
Professional development and support of midwifery students	Purple	7	Midwifery students (466), mentor (30), continuity of care (23), professional (17), experiences (8), support (7), workforce (7)	Mentoring processes of midwifery students, continuity of care, professional development and experiences, support systems and professional workforce
Development of clinical skills and simulation-based education	Turquoise	5	Clinical skills (105), simulation (55), competence (42), OSCE (9), confidence (7)	Development of clinical skills, simulation-based education

While the keywords in the first theme focus on improving the quality and standards of educational processes, the second theme reflects the research approaches and evolving educational agendas in the field of midwifery education. The third theme highlights curriculum development and innovative teaching approaches in the education of health professionals. The fourth theme represents assessment, collaboration, and communication processes that support professional development, while the fifth theme focuses on supporting students’ professional development. The sixth theme highlights the importance of simulation-based learning approaches in developing clinical competencies. When considered as a whole, the literature on midwifery education is structured around 4 main areas: education, research, professional development, and clinical practice (Table 4).

**Figure 7.**
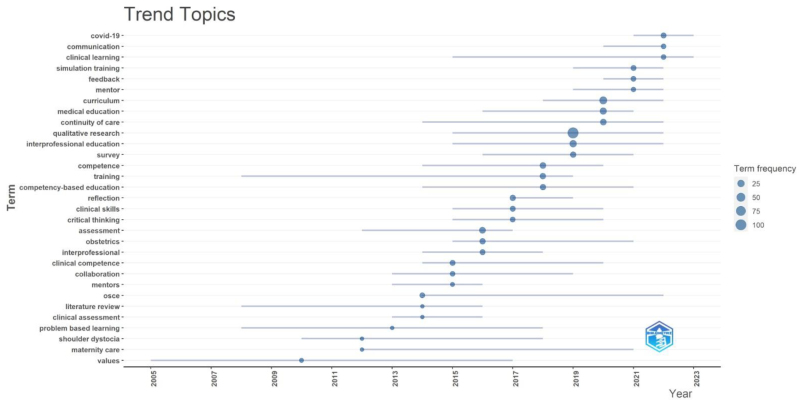
Trend topic analysis of author keywords.

Trend topics in midwifery education in recent years are presented in Figure 7. The size of the bubble indicates the frequency of the keyword use. The horizontal lines represent the time period during which the keyword appears in the literature. When Figure 7 is examined, it is understood that the topics of keywords such as values, problem-based learning, literature review, and training have been studied for a long time and have remained on the agenda for many years. When the trends in recent years are examined, it is seen that the topics of COVID-19, communication, clinical learning, simulation training, feedback, and mentoring come to the fore.

## 4. Discussion

This study has provided a comprehensive snapshot of the international literature on midwifery education by applying bibliometric methods to scientific publications, the basic and conceptual structure, achievements, and gaps of the current research field on midwifery education in the world. Thus, a detailed examination of the publications on midwifery education and the basic structure of this field contributes greatly to our understanding of the global trend in midwifery education in academic and practitioner contexts and how scientific innovations develop and evolve.

The study included and analyzed 1029 publications on midwifery education. Our analysis results show that the research literature in the field of midwifery education has expanded rapidly in the last decade. The increase in the midwifery education research literature may be associated with some main factors. First, it is believed that innovations in the field of health may be linked to an increase in research activities.^[19,20]^ In addition, it is evident that approaches such as digitalization, educational technologies, distance learning, and simulation-based education are increasingly featured in the literature, and this trend parallels the growing diversity of research.^[21]^ Moreover, the process by which midwifery education adapts to modern health needs and global health crises is a topic that has garnered increasing attention in the academic literature and offers important insights into the future direction of the field.^[19,20]^ Developments in educational technologies and applications such as distance education methods that are being adapted to the education system may contribute to the further expansion of research in the field of midwifery education in the future. In the meantime, our research findings show that most authors have only one publication on midwifery education. This finding may indicate that many researchers contribute to the field only occasionally rather than maintaining a sustained research focus on midwifery education.^[22]^

Our findings showed that the first 10 most cited articles were significantly dominated by qualitative research. The high citation rates of qualitative studies indicate that such studies deeply address the experiential and practical aspects of midwifery education and are taken into consideration by other researchers.^[23,24]^ Qualitative studies contribute to the enrichment of the literature in this field by providing important information in understanding the difficulties and successes experienced by practitioners and students.^[25,26]^ The high number of citations for simulation-based learning and continuity-based care models suggests that educational approaches aimed at developing clinical competence and supporting continuity of care in midwifery education have become a focus of academic interest. Simulation-based learning is recommended as an important tool to increase the professional confidence of midwife candidates.^[27]^ Simulation-based learning, additionally, has attracted attention in research in midwifery education as an innovative alternative to traditional approaches and as a bridge between theory and practice.^[28]^ The most cited research area, the continuous care model in midwifery, has become both a health policy on the agenda of different countries and an important component of quality standards in midwifery education. Furthermore, as continuous care models become more widespread, they may offer increasing opportunities for students to gain experience in providing continuity through placement within continuity models of care.^[29]^

The concentration of publications and citations in certain journals suggests that specialized journals in the field of midwifery education play a significant role in shaping the research agenda and disseminating knowledge. Studies support that these journals have expanded the knowledge base in the field by publishing important research on midwifery education and have created a high impact on academic literatüre.^[30,31]^ Moreover, since these journals will provide specific information on midwifery education, they can provide an important source of information for researchers, be a suitable platform for publication, and lead the growth of the midwifery education research field.

In midwifery education literature, inter-institutional productivity is of great importance in understanding which institutions conduct research activities and the scientific productivity of these institutions. In our study, Australian-centered universities stand out among the most productive institutions. Australia also ranked first among the countries with the most studies in the field of midwifery education. This shows the leadership of Australia and its institutions in the midwifery education literature. This finding also reflects the strong academic infrastructure and supportive research environment in the country.^[25]^

However, Australia dominant position in this field cannot be explained solely by research productivity. The early integration of continuity-based midwifery care models into educational programs, robust research funding mechanisms, and institutional support for academic midwifery research may be among the factors explaining this leadership.^[32,33]^ In contrast, the limited visibility of low- and middle-income countries can be interpreted as a reflection of inequalities in global knowledge production and disparities in research infrastructure.^[34,35]^ This situation highlights the need to make global evidence generation in midwifery education more inclusive and to increase the visibility of research from diverse geographical regions. In addition, the structure of international indexing systems can also affect the visibility of research produced in certain regions and exacerbate inequalities in global knowledge production.

Australia is also notable for its strong collaboration network with the United Kingdom. Australia and the United Kingdom have similar academic and practical approaches to midwifery education and practices. In addition, the high productivity and collaboration observed between these 2 countries may be due to joint research funding, academic networks, and the implementation of joint projects.^[32,33]^ Another country with a high number of collaborations was the United States. Collaborations in the 3 countries reflect high academic productivity as well as their capacity to share knowledge and compound resources internationally.^[34,35]^ The more limited collaboration networks in other countries may indicate resource constraints, limited research capacity, or a lack of international collaboration opportunities.

In the thematic review of the common word analysis, it was seen that the quality of education and training was the most researched theme. The fact that the quality of education has emerged as the dominant theme suggests that midwifery education focuses not only on the transfer of knowledge but also on the development of competencies aligned with international standards. This finding may reflect the growing emphasis on quality assurance and patient safety principles in healthcare systems in recent years, as well as the incorporation of these principles into educational programs. In this theme, issues such as the quality of educational processes in midwifery education, information management, and accreditation were at the forefront. This result shows that the World Health Organization goals of strengthening the quality of midwifery education for universal health coverage by 2030 were supported.^[4]^

The theme of “research in midwifery education” primarily covered emerging issues affecting research methods, data collection approaches, and educational practices. The prominence of topics such as ethics, the COVID-19 pandemic, and technology-enhanced learning indicates that research in midwifery education is increasingly responding to current challenges and transformations in the field of health education.^[36–38]^ These findings highlight the growing interest in the role of technology in midwifery education and demonstrate that digital learning environments may influence learning experiences, educational programs, and expected professional competencies. In particular, the widespread adoption of digital learning environments is expanding the scope of digital competencies expected of students and necessitates that educational programs adapt to this transformation.

The theme of “education and training of health professionals” covers issues such as curriculum development and interdisciplinary education. The prominence of this category also supports the outcome expressed by Kubato and his colleagues that educational curricula should be constantly updated and interdisciplinary collaboration should be increased.^[39]^ In our study, the trends in midwifery education in recent years were examined in detail, and it was concluded that values, problem-based learning, literature review, and training represent long-term research areas and are among the concepts that have been consistently included in the literature over the years. These results show the importance of an effective education process not only in the transfer of knowledge but also in the acquisition of professional values and the use of different teaching methods. Ben Nia et al also stated that standards and values should be reflected in order to increase the quality of education.^[40]^ Hmelo-Silver emphasized the importance of active learning methods in education and suggested that it is a well-established research area that improves students’ problem-solving skills.^[41]^ The fact that this topic encourages student-centered approaches in education and deepens learning processes explains its long-term impact on midwifery education literature.

Changes in the keywords used in these studies indicate that midwifery education research is closely linked to the global health agenda and innovative approaches in education. It is evident that the changes in health professions education during the COVID-19 pandemic have been reflected in the literature and have influenced the topics addressed in midwifery education research.^[42,43]^ This suggests that midwifery education research is a dynamic field shaped by global health issues and technological advancements. Additionally, there is a growing interest in digital competencies and technology-supported learning approaches in the literature.^[43,44]^ This shift in research topics contributes to understanding how current developments and new requirements in the field of health education are reflected in the academic literature.

## 5. Conclusion

This bibliometric analysis has comprehensively revealed the development, research priorities, and knowledge structure of the international literature in the field of midwifery education. The findings indicate that midwifery education research has grown significantly in recent years and has centered on education quality, professional development, clinical competence, and innovative teaching approaches. In particular, simulation-based learning, continuity of care, digital learning environments, and competency-based education approaches are gaining increasing prominence in the literature.

The study also revealed distinct differences in global knowledge production. While countries such as Australia, the United Kingdom, and the United States have made significant contributions to the field development, the limited representation of low- and middle-income countries in the literature highlights the need for more inclusive research collaborations and knowledge sharing on a global scale.

The findings offer important implications for updating curricula in midwifery education, strengthening digital and clinical competencies, and supporting quality-focused educational approaches. Additionally, the study provides an evidence-based framework for developing educational policies, strengthening quality assurance processes, and promoting international research collaborations. It is recommended that future research focus on the impact of AI-supported learning applications on educational outcomes, the development of digital literacy, and approaches that support more effective participation in knowledge production across different geographic and cultural contexts.

The findings of this study should be interpreted within a framework where the limitations that arise due to the nature of bibliometric analysis should be taken into consideration. The first limitation of this study is that it evaluated only publications indexed in the WoS database. While WoS is a robust and widely used resource for bibliometric studies, some studies found in Scopus, PubMed, CINAHL, Embase, and regional databases were not included in the scope of the research. This situation may have led to insufficient representation of publications produced in low- and middle-income countries that are not indexed by WoS. Therefore, it should be noted that the findings reflect not the entire global midwifery education literature, but only the portion indexed by WoS. Future studies can perform a more comprehensive analysis using a combination of different databases. Secondly, it should be noted that since the literature review is based on article titles, abstracts, and keywords, the results may not fully reflect the studies in the field. Future studies can increase the number of studies examined by conducting a more comprehensive search that includes all articles. However, considering that the number of publications included in our analysis is sufficiently high, we believe that our findings reflect the general status and relevant trends in this field.

## Author contributions

**Conceptualization:** Nuray Kurt, Esra Sabanci Baransel.

**Formal analysis:** Nuray Kurt, Osman Tayyar Çelik, Esra Sabanci Baransel, Tuba Uçar.

**Investigation:** Nuray Kurt, Osman Tayyar Çelik, Esra Sabanci Baransel, Tuba Uçar.

**Methodology:** Nuray Kurt, Osman Tayyar Çelik, Esra Sabanci Baransel, Tuba Uçar.

**Writing – original draft:** Osman Tayyar Çelik, Esra Sabanci Baransel, Tuba Uçar.

**Writing – review & editing:** Osman Tayyar Çelik, Tuba Uçar.
